# Mycophenolate mofetil and tacrolimus versus tacrolimus alone for the treatment of idiopathic membranous glomerulonephritis: a randomised controlled trial

**DOI:** 10.1186/s12882-019-1539-z

**Published:** 2019-09-06

**Authors:** Aikaterini Nikolopoulou, Marie Condon, Tabitha Turner-Stokes, H. Terence Cook, Neill Duncan, Jack W. Galliford, Jeremy B. Levy, Liz Lightstone, Charles D. Pusey, Candice Roufosse, Thomas D. Cairns, Megan E. Griffith

**Affiliations:** 1 Centre for Inflammatory Disease, Division of Immunology and Inflammation, Department of Medicine, Imperial College London, Du Cane Road, London, W12 0NN UK; 2 Imperial College Renal and Transplant Centre, Imperial College Healthcare NHS Trust, Hammersmith Hospital, London, W12 0NN UK

**Keywords:** Membranous nephropathy, Nephrotic syndrome, Relapse, Mycophenolate mofetil, Tacrolimus, Randomised controlled trial

## Abstract

**Background:**

Tacrolimus (TAC) is effective in treating membranous nephropathy (MN); however relapses are frequent after treatment cessation. We conducted a randomised controlled trial to examine whether the addition of mycophenolate mofetil (MMF) to TAC would reduce relapse rate.

**Methods:**

Forty patients with biopsy proven idiopathic MN and nephrotic syndrome were randomly assigned to receive either TAC monotherapy (*n* = 20) or TAC combined with MMF (*n* = 20) for 12 months. When patients had been in remission for 1 year on treatment the MMF was stopped and the TAC gradually withdrawn in both groups over 6 months. Patients also received supportive treatment with angiotensin blockade, statins, diuretics and anticoagulation as needed. Primary endpoint was relapse rate following treatment withdrawal. Secondary outcomes were remission rate, time to remission and change in renal function.

**Results:**

16/20 (80%) of patients in the TAC group achieved remission compared to 19/20 (95%) in the TAC/MMF group (*p* = 0.34). The median time to remission in the TAC group was 54 weeks compared to 40 weeks in the TAC/MMF group (*p* = 0.46). There was no difference in the relapse rate between the groups: 8/16 (50%) patients in the TAC group relapsed compared to 8/19 (42%) in the TAC/MMF group (*p* = 0.7). The addition of MMF to TAC did not adversely affect the safety of the treatment.

**Conclusions:**

Addition of MMF to TAC does not alter the relapse rate of nephrotic syndrome in patients with MN.

**Trial registration:**

This trial is registered with EudraCTN2008–001009-41. Trial registration date 2008-10-08.

## Background

Membranous nephropathy (MN) is a relatively common cause of the nephrotic syndrome in adults affecting 5–10 per million population per year. Although spontaneous remissions are common, 40% of patients with untreated membranous nephropathy will develop end stage renal disease within 10–15 years [1].

Treatment of idiopathic membranous nephropathy can be challenging. The discovery of anti-phospholipase A2 receptor (anti-PLA2R) antibodies in 70% of patients with MN [2] supports the use of immunosuppression for the treatment of idiopathic MN. Treatment regimens including high dose steroids with chlorambucil or cyclophosphamide have proven to be effective in inducing remission [3, 4] however these regimens have significant adverse effects [5] and relapses are not infrequent.

When this trial was designed there were limited data on the use of rituximab in MN and only a small case series of 8 patients showed an effect of the agent on proteinuria [6]. Since then rituximab has gained popularity following data from clinical trials that support its use in MN [6–9].The immunological effect of the agent was confirmed in an initial study that showed that anti-PLA2R antibodies disappeared in 68% (17 out of 25) of patients with MN treated with rituximab [10]. In addition, more recently both the GEMRITUX and the MENTOR trials showed that rituximab is effective in inducing remission of idiopathic MN [7, 8]. However there are significant numbers of patients who are resistant to rituximab and its use is still restricted in many centres due to cost. Therefore alternative treatment options are necessary and other agents used include calcineurin inhibitors and mycophenolate mofetil.

Calcineurin inhibitors (CNIs) such as cyclosporin and tacrolimus (TAC) are commonly used for the treatment of MN and are effective in inducing remission. Their antiproteinuric effect has been clearly documented [11–13]; however relapses after discontinuation are common and there is a concern about nephrotoxicity after prolonged use. Although their main mode of action is immunomodulatory they also have a significant antiproteinuric effect which is attributed to their haemodynamic effect on glomerular perfusion as well as to their direct effect on podocytes [12, 14]. A study comparing tacrolimus to placebo showed that TAC alone is effective in achieving remission but patients often relapse after treatment withdrawal [15]. A recent trial comparing rituximab to cyclosporine showed a high relapse rate of the cyclosporine arm after treatment withdrawal [8].

Mycophenolate mofetil (MMF) has been used for the treatment of MN in regimens of variable dosing and duration, either alone or in combination with steroids [16–18]. MMF monotherapy is not effective in inducing remission [17]; however it has been successfully used in combination with steroids [16, 19]. MMF has a long history of use in combination with CNIs in transplantation with a good safety profile and this combination has also been used in nephrotic syndrome secondary to other glomerulonephritides such as lupus nephritis [20, 21]. The presence of anti-PLA2R auto-antibodies in a significant number of patients with MN suggests that an immunosuppressive agent such as MMF, that suppresses B lymphocyte proliferation and antibody formation, may be particularly effective in inducing and maintaining immunological and clinical remission in MN.

We compared the efficacy of combination treatment with tacrolimus and mycophenolate mofetil versus tacrolimus alone for achieving sustained remission in patients with idiopathic membranous nephropathy.

## Methods

### Trial design and participants

In this single centre randomised controlled trial patients with biopsy proven primary membranous nephropathy were recruited to receive tacrolimus in combination with mycophenolate mofetil or tacrolimus alone. Inclusion criteria were: patients 18–80 years old; idiopathic membranous nephropathy on renal biopsy and with no evidence of an underlying cause; proteinuria defined by urinary protein: creatinine ratio (uPCR) > 100 mg/ mmol with hypoalbuminaemia defined by serum albumin < 35 g/l or uPCR > 300 mg/ mmol with normal serum albumin, despite 3 months treatment with maximum tolerated dose of angiotensin enzyme inhibitors (ACEI) or angiotensin II receptor blockers (ARB).

We excluded patients with membranous nephropathy secondary to other causes: positivity for Hepatitis B, C or HIV; malignancy; untreated infection. We also excluded pregnant or breastfeeding females and those planning a pregnancy or using unreliable contraception.

All patients were screened for malignancy with CT scan of the chest, abdomen and pelvis.

Eligible patients were randomly assigned to receive either tacrolimus (TAC group) or tacrolimus in combination with mycophenolate mofetil (TAC/MMF group). All patients remained on supportive therapy including ACEI and ARB, diuretics, statins and anticoagulation. Those assigned to the TAC group received an initial dose of 2 mg twice daily titrated to achieve whole blood levels of 5–12 ng/ml. Those assigned to the TAC/MMF group also received MMF 500 mg twice daily titrated to achieve blood mycophenolic acid (MPA) levels of 1.5–3.0 mg/L.

Patients were initially treated for 1 year. Remission of proteinuria was defined as complete (CR) when uPCR was less than 30 mg/mmol and partial (PR) if uPCR decreased by more than 50% but remained above 30 mg/mmol and less than 300 mg/mmol. Patients not obtaining complete or partial remission within 12 months were withdrawn from the trial. Once patients were in remission for 12 months the MMF was stopped in the TAC/MMF group and tacrolimus gradually withdrawn over 6 months in both groups. Maximum duration of therapy prior to treatment withdrawal was 24 months.

Patients were seen weekly until stable and established on adequate levels of immunosuppressive agents, and then monthly until remission achieved. Blood and urine samples were collected at each visit. Full blood count, biochemical profile, drug levels and proteinuria were measured. The study was conducted via the glomerulonephritis clinic at the Imperial College Healthcare NHS Trust, Hammersmith hospital, London. Data were collected in paper case report forms and progress of the trial was reported to and monitored by the West London Renal and Transplant Glomerulonephritis Research Group at Hammersmith Hospital, on a monthly basis.

Randomisation was performed by the clinical coordinating team participating in the trial; allocation concealment was performed by enclosing assignments in sequentially numbered, opaque, sealed envelopes.

The trial was sponsored by the Imperial College Healthcare NHS Trust and supported by the National Institute for Health Research Imperial Biomedical Research Centre. The trial was monitored by the Joint Research Compliance Office. Ethics approval was obtained by the London Hampstead REC. Trial registration: EudraCT Number 2008–001009-41.

### End points

The primary end point was the efficacy of MMF in preventing relapse of the nephrotic syndrome 6 months after withdrawal of tacrolimus therapy. Secondary end points were time to remission, degree of remission (complete or partial) and change in renal function.

### Sample size

The sample size was based on the primary end point, time to relapse, and compared using a 1-sided log rank test assuming a 1% drop out rate and a 50% survival rate in the tacrolimus group and a 95% survival rate in the tacrolimus and MMF group with an alpha level of 0.05 and 80% power. The initial sample size was 32; this was increased to 40 in 2013 by a substantial amendment to the trial approved by the London Hampstead REC and MHRA to compensate for the higher than expected dropout rate.

### Statistical analysis

Continuous data are reported as median; categorical data are presented as absolute values or percentages. Categorical variables were analysed using chi-squared test or Fisher’s exact test where appropriate. A *P* value of < 0.05 was considered as statistically significant. Statistical analyses were performed using PRISM statistical software. The Imperial College Statistical Advisory Service oversaw the statistical analysis.

## Results

**Fig. 1:**
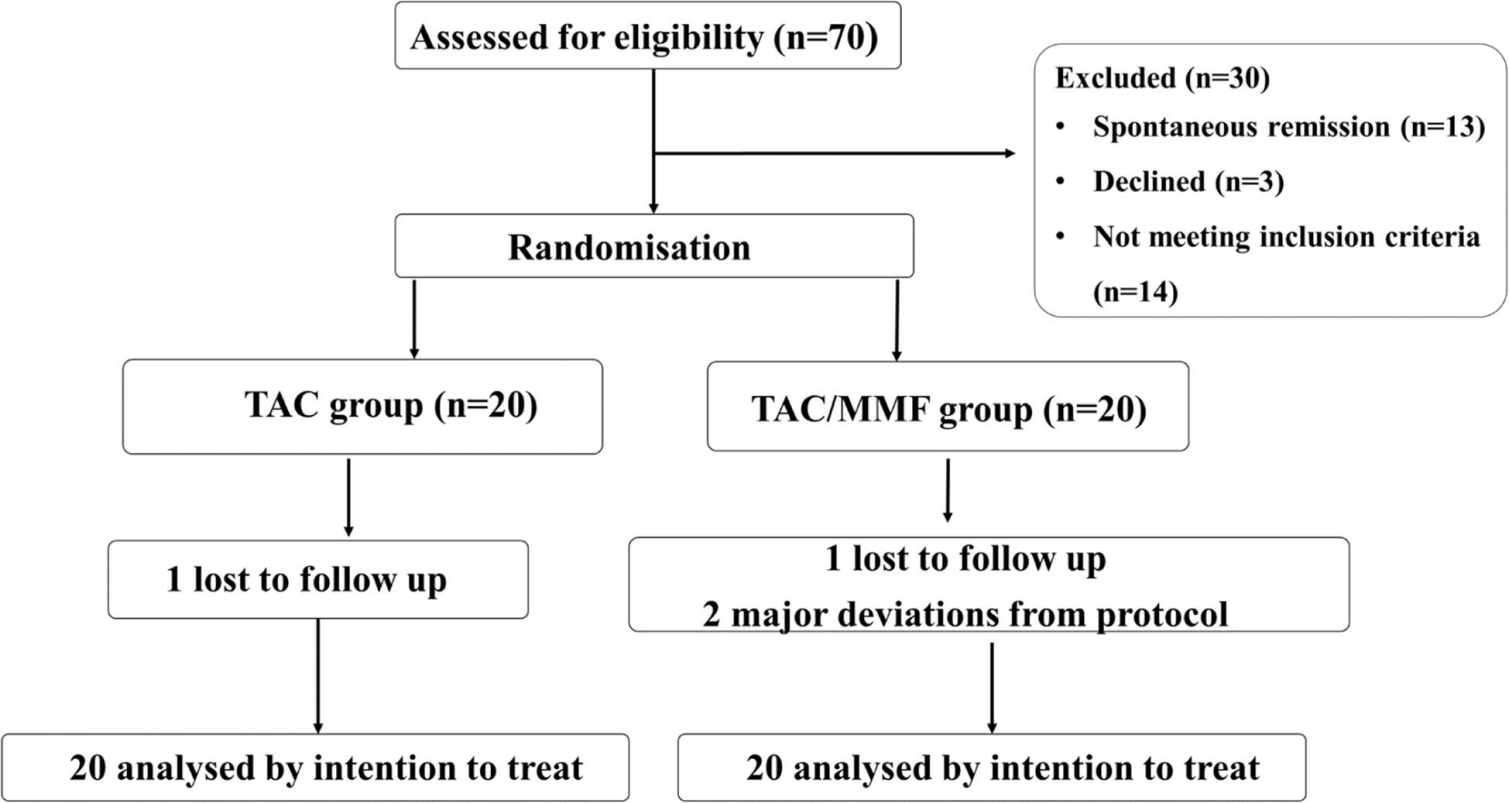
Trial profile. In the TAC/MMF group the 2 major deviations from protocol involved one patient who declined to start the MMF and one who travelled abroad and stopped the allocated trial medication

Forty Patients were recruited to the trial between March 2009 and December 2014. 20 patients were randomly assigned to receive TAC alone and 20 patients to combination treatment with TAC and MMF (Fig. 1). The groups had similar baseline characteristics (Table 1). 39 out of 40 patients had a new diagnosis of MN. One patient in the TAC group had received previous treatment for membranous nephropathy with steroids and chlorambucil 10 years prior to entering the trial. This patient had since been off treatment and was recruited to the trial during a relapse.

**Table 1:** Demographic, laboratory and histological characteristics of patients at baseline

	TAC	TAC/MMF
*Age yr (median - range)*	55 (24–68)	48 (28–66)
*Gender*		
Male/female	11/9	13/7
*Ethnicity*		
Asian	8	7
Afro-Caribbean	3	4
Caucasian	9	9
*Previous treatment with IS*	1	0
*SCr mg/dl*	0.8 (0.5–1.4)	0.8 (0.5–1.2)
*Estimated GFR mls/min/1.73m* ^*2*^ *(median-range)*	109 (44–142)	121 (63–201)
*UPCR mg/mmol (median-range)*	704 (203–2159)	756 (123–1784)
*Serum Albumin g/L (median-range)*	17 (8–30)	18 (11–27)
*Systolic BP mmHg (median-range)*	119 (101–155)	131 (110–179)
*Diastolic BP mmHg (median-range)*	77.5 (65–88)	81 (66–119)
*ACEI and/or ARB*	20/20	17/20
*Histological stage of deposits at biopsy I/II/IIII/IV*	4/12/3/3	3/12/3/5
*PLA2R pos/neg/unknown*	10/8/2	12/7/1
IFTA 0/ 5–10%/ 10–20%	10/10/0	7/11/2

One patient in the TAC group received previous treatment with steroids and chlorambucil.

*Abbreviations*: *IS* immunosuppression, *SCr* serum creatinine, *GFR* glomerular filtration rate calculated by the 4 variable Modification of Diet in Renal Disease, *UPCR* urinary protein creatinine ratio, *ACEI* angiotensin converting enzyme inhibitor, *ARB* angiotensin receptor blocker, *PLA2R* phospholipase A2 receptor, *IFTA* interstitial fibrosis and tubular atrophy

11/20 patients in the TAC group and 13/20 in the TAC/MMF group were male. Baseline uPCR and renal function were equivalent in the two groups. In the TAC group median uPCR and serum creatinine at baseline were 704 mg/mmol (range 203–2159) and 0.8 mg/dl (range 0.5–1.4) respectively; in the TAC/MMF group median uPCR and serum creatinine were 756 mg/mmol (range 123–1784) and 0.8 mg/dl (range 0.5–1.20) respectively. All (20/20) patients in the TAC group and 17/20 patients in the TAC/MMF group were on an ACEI/ARB or a combination of both at the start of trial and for at least 3 months prior to enrolment; three patients in the TAC/MMF group were intolerant to ACEI/ARB due to hypotension or cough. The age, morphology and location of the electron dense deposits were recorded by electron microscopy. 12/20 biopsies in each group were histological stage II. PLA2R positivity on biopsy was detected in 10/20 patients in the TAC group and 12/20 in the TAC/MMF group. Interstitial fibrosis and tubular atrophy (IFTA) was significant (15%) in 2 cases in the TAC/MMF group but less than 10% in the rest of the biopsies at baseline. Demographic, laboratory and histological characteristics at baseline are shown on Table 1.

The median follow up in the TAC group was 6 years (range 6 months to 8.8 years) compared to 5.9 years (range 9 months to 8.8 years) in the TAC/MMF group, including patients that were lost to follow up early during the treatment phase.

### End points

**Fig. 2:**
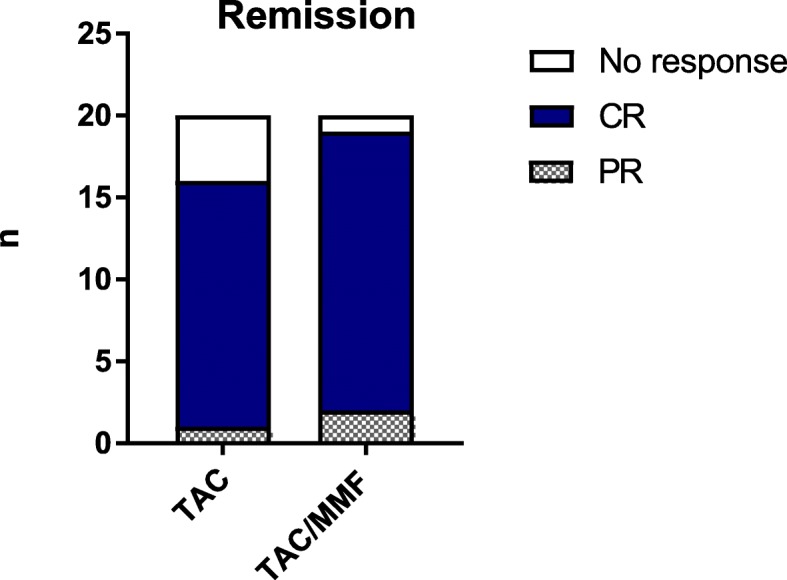
Complete (CR) and partial remission (PR) in the two groups

In the TAC group 16/20 (80%) achieved remission (both PR and CR) compared to 19/20 (95%) in the TAC/MMF group (*p* = 0.34) (Fig. 2). One patient in the TAC group and two in the TAC/MMF group reached PR only. The median time to remission (CR/PR) in the TAC group was 54 weeks compared to 40 weeks in the TAC/MMF group, suggesting a possible trend to earlier remission in the TAC/MMF group, but this was not statistically significant (*p* = 0.46). The four patients in the TAC group and the one in the TAC/MMF group that did not achieve remission were withdrawn from the trial and alternative treatment given.

**Fig. 3:**
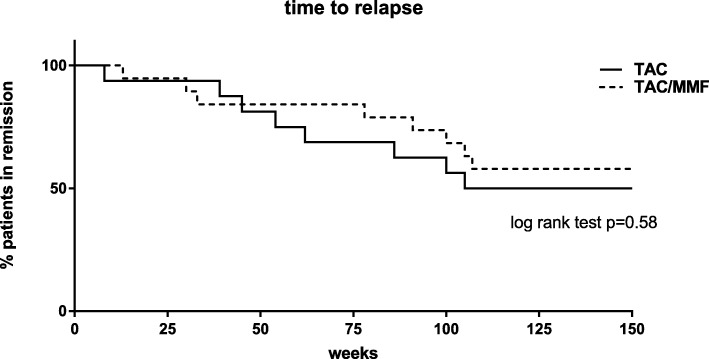
Time to relapse from commencement of treatment withdrawal. Relapse events occurred within 2 years from treatment withdrawal

There was no difference between the groups in the number of patients who subsequently relapsed. In the TAC group 8/16 patients (50%) relapsed and in the TAC/MMF group 8/19 patients (42%) relapsed (*p* = 0.7). In the TAC group two patients relapsed during withdrawal and six after stopping TAC (median time to relapse after stopping was 40 weeks, range 10–67); in the TAC/MMF group four patients relapsed during withdrawal and four after stopping TAC (median time to relapse after stopping TAC was 45 weeks (range 25–109), (log rank test *p* = 0.58) (Fig. 3).

**Fig. 4:**
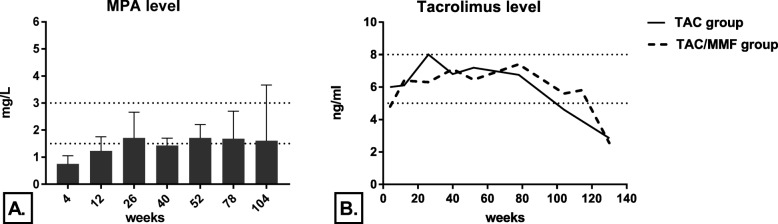
**a** Median mycophenolic acid (MPA) level. **b** Median tacrolimus levels in the two groups

MPA levels and tacrolimus levels were within target range (1.5–3.0 mg/L and 5–8 ng/ml respectively) in the two groups (Fig. 4).

A non-statistically significant rise in the serum creatinine from baseline to 2 years was noted in both treatment groups. In the TAC group the serum creatinine rose from 0.8 mg/dl (median; range 0.5–1.4) to 1 mg/dl (median; range 0.7–2.4) at 2 years (*p* = 0.9); there was no further deterioration and at 4 years it remained at this level (1.05 mg /dl, median; range 0.7–1.8) (*p* = 0.9). Equally in the TAC/MMF group there was a non-statistically significant rise in the serum creatinine from baseline to 2 years from 0.8 mg/dl (median; range 0.5–1.2) to 1.15 mg/dl (median; range 0.9–1.5) (*p* = 0.9) and it remained at this level at 4 years (1.1 mg/dl, median; range 0.7–2.3) (*p* = 0.9). In the TAC group two patients developed CNI toxicity on biopsy and were subsequently withdrawn from the trial. Histopathological examination of their renal biopsy showed increased IFTA by 20 and 30% respectively from baseline and arteriolar hyalinosis consistent with nephrotoxicity from CNI. End stage renal disease developed in two other patients, one from each group, who failed to respond to the trial treatment and were withdrawn. The patient from the TAC group subsequently received steroids and cyclophosphamide however required haemodialysis 8 months later and the patient from the TAC/MMF group was treated with rituximab but required dialysis 2 years later. Both these patients were males over 60 years old with similar serum creatinine at baseline (0.7 and 0.8 mg/dl respectively).

**Fig. 5:**
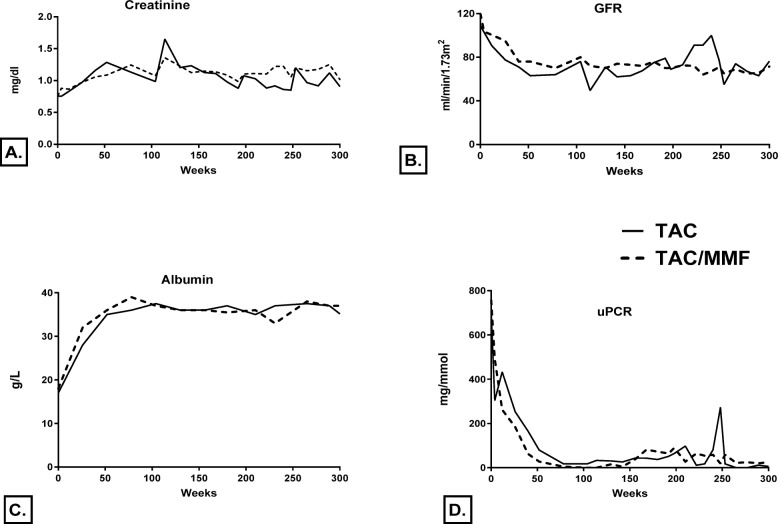
**a** Serum creatinine (mg/dl, excluding patients requiring dialysis). **b** Glomerular filtration rate in ml/min/1.73m^2^ *(*excluding patients requiring dialysis) **c** Serum albumin g/l **d**: Urinary protein creatinine ratio (mg/mmol) Note: To convert serum creatinine from mg/dl to μmol/L, multiply by 88.4; estimated glomerular filtration rate calculated by the 4 variable Modification of Diet in Renal Disease study

Median serum albumin levels increased by 6 months and normalised by 12 months in both groups. In the TAC group serum albumin increased from 17 g/L (median; range 8–30) to 26 g/L (median; range 13–36) at 6 months and to 35 g/L (median; range 17–40) at 12 months (*p* < 0.001). In the TAC/MMF group serum albumin improved from 18 g/L (median, range 11–27) to 32 g/L (median; range 18–40) at 6 months and to 36 g/L (median; range 25–42) at 12 months (*p* < 0.001). This improvement was accompanied by the reduction of median uPCR by more than 50% by 6 months in both groups. In the TAC group median uPCR decreased from 704 mg/mmol (median; range 203–2159) at baseline to 253 mg/mmol (median; range 0–1128) at 6 months and to 79 mg/mmol (median; range 0–1142) at 12 months (*p* < 0.001). In the TAC/MMF group median uPCR reduced from 756 mg/mmol (median; range 123–1784) at baseline to 184 mg/mmol (median; range 22–522) at 6 months with a further reduction to 27 mg/mmol (median; range 0–217) at 12 months (*p* < 0.001). Serum creatinine, GFR, albumin and proteinuria levels over time are shown in Fig. 5.

### Adverse events

We recorded a total of 18 serious adverse events (SAE) in both groups. 7 patients in the TAC group experienced a total of 12 SAEs and 3 in the TAC/MMF group experienced a total of 6 SAEs. In the TAC group 3/12 SAEs were possibly related to the trial drug, the rest were not related (6/12) or unlikely to be related (3/12). None of the 6 SAEs occurring in the TAC/MMF group were related to the trial medication. A list of the serious adverse events observed in the two groups is presented in Table 2. These include all serious events that required hospitalisation irrespective of their relation to the trial medication.

**Table 2:** Serious adverse events (SAE) by treatment group

	TAC	TAC/MMF
Patients with at least 1 SAE	7	3
Total number of SAEs	12	6
Diarrhoea/vomiting	3	2
High INR/Bleeding/Anaemia		2
Haemorrhoidectomy		1
Haematuria/ Urinary tract infection	2	
Blackout	1	
AKI	3	
Headache	1	
Infection	1	
Gouty arthritis	1	
Cholestatic jaundice		1

In the TAC group 3/12 SAEs were possibly related to trial drug, 3/12 were unlikely to be related to trial drug and the rest (6/12) were not related to trial drug. None of the 6 SAEs reported in the TAC/MMF group were related to trial drugs.

*AKI* acute kidney injury

## Discussion

Idiopathic MN is a common cause of the nephrotic syndrome in adults and untreated can lead to ESRD in up to 40% of patients within 10 years. The role of immunosuppression including biological agents in the management of MN is clearer now than when this trial started more than 10 years ago; however the optimal choice of agent remains controversial. In one small pilot study MMF was successfully used in a cohort of patients with MN resistant to steroids and where cytotoxics and cyclosporine failed [22]. In this study patients achieved significant reduction in proteinuria within 6 months of treatment with MMF monotherapy. However a more comprehensive study of 36 patients with idiopathic MN, treated with MMF or conservative treatment for 12 months, showed no benefit of MMF monotherapy in inducing remission compared to the control group receiving supportive care only [17]. Other randomised trials have shown a benefit from MMF when used in combination with steroids [16, 18, 19]; Chan et al. reported similar efficacy of MMF and steroids when compared to a modified Ponticelli regimen using chlorambucil [19], and Brantel et al. showed that a 12 month course of MMF and prednisolone was as effective as cyclophosphamide in decreasing proteinuria when compared to a historic group treated with the Ponticelli regimen [16]. Side effects from steroids are a concern however, and given the apparent effectiveness of MMF in MN when used in combination with other immunosuppressants, it was hypothesised in this trial that MMF may reduce the relapse rate of nephrotic syndrome when used in combination with TAC for the treatment of MN.

However this study does not show a benefit of MMF when added to TAC in reducing relapse rate. The trend of the TAC/MMF group to achieve remission sooner than the TAC alone group did not reach statistical significance but it perhaps suggests that in some patients the addition of MMF to TAC may have some effect in achieving effective remission faster. However the numbers are small and the trial was only powered to look at relapse rates. When our study was designed, anti-PLA2R antibodies had not yet been discovered; hence we did not include anti-PLA2R levels in our assessment or end points. It is now known that anti-PLA2R levels are associated with disease activity and outcomes [23, 24]. We were able to retrospectively stain the available biopsies for PLA2R and this was found to be positive in 55 and 63% of patients in the TAC and the TAC/MMF group respectively. The percentage of anti-PLA2R positivity in our cohort is lower than in some other studies and perhaps this has masked some benefit of the MMF. It is possible that patients with high antibody titres, which are associated with resistant disease, could benefit more from the addition of MMF than those with a low or absent titre. In our study, 4 patients in the TAC group compared to only 1 in the TAC/MMF group did not achieve remission and although this was not statistically significant it is also possible that the addition of MMF to TAC may be of benefit to some patients. Again the availability of anti-PLA2R levels would help interpret these results and larger studies with specific subgroups are required to further investigate treatment options.

Importantly the combination of MMF with TAC appeared to be safe in this group of patients, with only 3 patients experiencing a serious adverse event in the TAC/MMF group. Infection and leukopenia were not a major issue in the combination arm. A novelty in our study was to monitor MPA levels during treatment for MN; MMF dose was titrated to achieve MPA levels between 1.5–3 mg/ml to ensure effective treatment and minimise side effects, and this may have contributed to the safety of the combination therapy, although nephrotoxicity due to CNI was an issue in 2 patients in the TAC group. Both these patients underwent renal biopsy for rising serum creatinine that showed tubular injury and hyalinosis consistent with CNI toxicity. In both treatment groups there was a non-significant rise in the serum creatinine from baseline to 2 years; in patients that were followed up for longer, the serum creatinine subsequently remained unchanged over time. Two patients progressed to end stage renal disease despite treatment with the trial medication or other therapies, and this demonstrates that existing treatments are not always effective, and more options should be available.

Recruitment was slow and the trial was designed over 10 years ago, without the current knowledge for the role of anti-PLA2R antibodies in MN or for the use of rituximab as first line treatment. Although randomised controlled trials (RCTs) are the most acknowledged methods of testing a treatment for a specific condition there are only a limited number of RCTs examining the efficacy of TAC alone in MN [15]. Therefore we believe that this RCT is important and the results relevant because they contribute to the evidence for the use of TAC and MMF as treatments for MN. Reporting a negative outcome is important in order to avoid using unnecessary or ineffective immunosuppression. The length of follow up is one of the strong points of the trial, and although this trial did not show a benefit from the addition of MMF to TAC it showed that TAC alone or in combination with MMF is safe in MN and CNI toxicity occurred in only two patients.

## Conclusions

In summary, this study did not show a benefit from the addition of MMF to TAC for the treatment of MN either in rates of remission, time to remission or subsequent relapse. Although this study is relatively small it was appropriately powered to detect a benefit of MMF on relapse rate. TAC is effective in inducing remission and it is well tolerated in most patients but CNI toxicity does occur therefore close monitoring of patients is required. Although effective regimens for the treatment of MN are available there are still patients who do not respond or are unable to receive specific biological or cytotoxic agents; therefore it is possible that the combination of TAC with MMF could allow lower doses of tacrolimus to be used to achieve remission and this should also be investigated. Most patients who relapsed did so after discontinuation of treatment rather than during withdrawal; therefore it could be that low doses of tacrolimus may be adequate to maintain remission once this has been achieved. Both treatment arms were well tolerated and further studies are needed to investigate if the combination of TAC/MMF would benefit a subgroup of patients with a high anti-PLA2R antibody titre and more resistant disease. Anti-PLA2R antibodies need to be included in future studies and comparative studies between the anti-PLA2R positive and negative patients would be useful to further clarify which patients may benefit from specific regimens so that immunosuppression can be better tailored to individual patients.

## Data Availability

The trial protocol and datasets used and/or analysed during the current study are available from the corresponding author on reasonable request.

## Abbreviations

ACE: Angiotensin enzyme inhibitors
ARB: Angiotensin II receptor blockers
CNI: Calcineurin inhibitor
CT: Computed tomography
HIV: Human immunodeficiency virus
IFTA: Interstitial fibrosis and tubular atrophy
MMF: Mycophenolate mofetil
MN: Membranous nephropathy
MPA: Mycophenolic acid
PCR: Protein creatinine ratio
PLA2R: Phospholipase A2 receptor
SAE: Serious adverse event
TAC: Tacrolimus
